# Predicting post-COVID-19 condition in children and young people up to 24 months after a positive SARS-CoV-2 PCR-test: the CLoCk study

**DOI:** 10.1186/s12916-024-03708-1

**Published:** 2024-11-07

**Authors:** Manjula D. Nugawela, Terence Stephenson, Roz Shafran, Trudie Chalder, Emma Dalrymple, Tamsin Ford, Lana Fox-Smith, Anthony Harnden, Isobel Heyman, Shamez N. Ladhani, Kelsey McOwat, Ruth Simmons, Olivia Swann, Elizabeth Whittaker, CLoCk Consortium, Bianca De Stavola, Esther Crawley, Kishan Sharma, Marta Buszewicz, Michael Levin, Shruti Garg, Vanessa Poustie, Snehal M. Pinto Pereira

**Affiliations:** 1 UCL Great Ormond Street Institute of Child Health, 30 Guilford Street, London, WC1N 1EH UK; 2 Department of Psychological Medicine, Institute of Psychiatry, Psychology and Neuroscience, King’s College London, De’Crespigny Park, London, SE5 8AF UK; 3 Department of Psychiatry, University of Cambridge, Hershel Smith Building Cambridge Biomedical Campus, Cambridge, CB2 0SZ UK; 4 Nuffield Department of Primary Care Health Sciences, University of Oxford, Oxford, OX2 6GG UK; 5 Immunisations and Vaccine Preventable Diseases, UK Health Security Agency, 61 Colindale Avenue, London, NW9 5EQ UK; 6 Centre for Paediatric and Neonatal Infection, St. George’s University of London, Cranmer Terrace, London, SW17 0RE UK; 7 Centre for Medical Informatics, Usher Institute, University of Edinburgh, Edinburgh, EH16 4TL UK; 8 Department of Paediatric Infectious Diseases, Imperial College Healthcare NHS Trust, London, W2 1NY UK; 9 Division of Surgery & Interventional Science, Faculty of Medical Sciences, University College London, London, WC1E 6BT UK

**Keywords:** Children and young people, Post-COVID-19 condition, Prediction model, Cohort study

## Abstract

**Background:**

Predicting which children and young people (CYP) are at the highest risk of developing post-COVID-19 condition (PCC) could improve care pathways. We aim to develop and validate prediction models for persistent PCC up to 24 months post-infection in CYP.

**Methods:**

CYP who were PCR-positive between September 2020 and March 2021, with follow-up data up to 24-months post-infection, were analysed. Persistent PCC was defined in two ways, as PCC at (a) 3, 6, 12 and 24 months post-infection (*N* = 943) or (b) 6, 12 and 24 months post-infection (*N* = 2373). Prediction models were developed using logistic regression; performance was assessed using calibration and discrimination measures; internal validation was performed via bootstrapping; the final model was adjusted for overfitting.

**Results:**

While 24.7% (233/943) of CYP met the PCC definition 3 months post-infection, only 7.2% (68/943) continued to meet the PCC definition at all three subsequent timepoints, i.e. at 6, 12 and 24 months. The final models predicting risk of persistent PCC (at 3, 6, 12 and 24 months and at 6, 12 and 24 months) contained sex (female), history of asthma, allergy problems, learning difficulties at school and family history of ongoing COVID-19 problems, with additional variables (e.g. older age at infection and region of residence) in the model predicting PCC at 6, 12 and 24 months. Internal validation showed minimal overfitting of models with good calibration and discrimination measures (optimism-adjusted calibration slope: 1.064–1.142; C-statistic: 0.724–0.755).

**Conclusions:**

To our knowledge, these are the only prediction models estimating the risk of CYP persistently meeting the PCC definition up to 24 months post-infection. The models could be used to triage CYP after infection. CYP with factors predicting longer-term symptomology, may benefit from earlier support.

**Supplementary Information:**

The online version contains supplementary material available at 10.1186/s12916-024-03708-1.

## Background

Post-COVID-19 condition (PCC), also known as long COVID, is difficult to research with many additional challenges compared to classic epidemiological studies [1]. In England, by June 2022, over 80% of 5-to-18-year-old children had antibodies against SARS-CoV-2, the virus responsible for COVID-19 and the recent pandemic. This likely reflects a combination of widespread (asymptomatic and symptomatic) infection and vaccination. For example, between January and March 2021, prior to vaccination rollout to children and young people (CYP), 85,546 CYP in England had a positive SARS-CoV-2 PCR test [2]. By July 2022, 62.4% of pupils aged 12 to 15 years and 80.5% of pupils aged 16 to 17 years at the start of the 2021/2022 academic year had received at least one dose of a coronavirus (COVID-19) vaccine, 45.3% and 69.8%, respectively, had received at least two doses [3]. What has also become clear is that some CYP report persistent symptoms months after acute SARS-CoV-2 infection, even if they were asymptomatic or had a low symptom burden at the time of infection [4–6]. Thus, being able to accurately predict PCC is valuable in order to identify those at highest risk and direct them towards relevant care. Such triaging is particularly relevant post-pandemic when health services are under unprecedented pressure [7].

Two particular challenges associated with researching PCC include the potential waxing and waning of symptoms after infection and the lack of a comparator group of CYP who have never been positive for SARS-CoV-2, due to widespread infection, especially since the emergence of highly transmissible variants. The CLoCk study [8] is a longitudinal cohort of SARS-CoV-2 PCR-positive and matched test-negative CYP, PCR-tested between September 2020 and March 2021 when they were aged 11 to 17 years. Using this data we have previously shown that, whilst the overall prevalence of PCC remains broadly stable up to 12 months post-infection, many CYP are classified as meeting the research definition of PCC [9] for the first time at 6 or 12 months post-infection [10, 11]. Therefore, examining PCC longitudinally and identifying those who persistently meet the PCC research definition is important in terms of characterising and predicting those who are likely to be impacted over a long time-period. We have also previously developed and validated a model to predict PCC in CYP 3 months after PCR testing [12]. In that analysis, we included both PCR test-positive and test-negative CYP and examined predictors of meeting our published consensus PCC research definition once (i.e. at 3 months post-testing) [9]. However, as we can no longer be certain that the original test-negative group remains uninfected, our new analyses will be restricted to the original test-positive group of CYP who were infected when the wild type and Alpha (B.1.1.7) variants were dominant.

Thus, using data from the CLoCk study [8] original test-positive group, we address two broad aims:

1. To describe the characteristics of CYP infected with the wild type or Alpha variants who persistently meet (vs. do not persistently meet) the PCC research definition over a 24-month period post-infection. Specifically, we describe these characteristics in the CLoCk sample who met the PCC research definition at 3, 6, 12 and 24 months post-infection (and similarly in a supplementary analysis, those who met the PCC research definition at 6, 12 and 24 months post-infection).
2. To develop and validate prediction models for persistent PCC in CYP up to 24 months post-infection.

## Methods

**Fig. 1:**
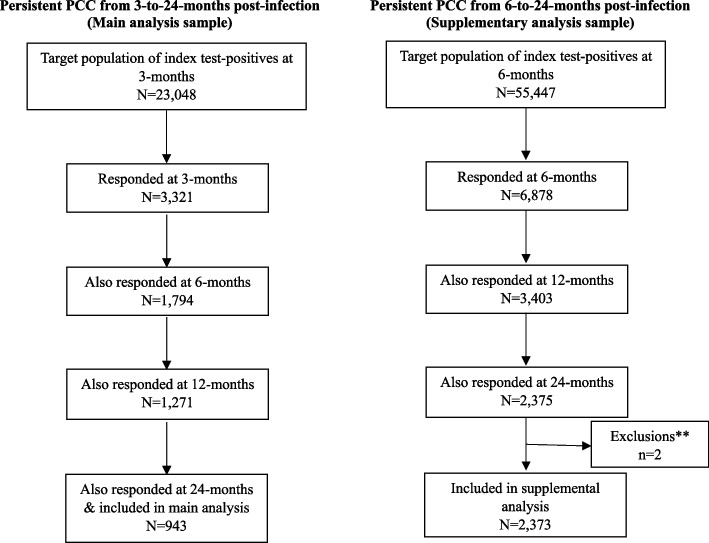
Data flow diagrams for the analytical samples under consideration*. *For a detailed overview of the CLoCk sampling strategy see *Nugawela *et al*. Int J Epidemiol 2024 53(1)*; ** we excluded 2 CYP from the final analytical sample because they had missing data on one of two questions: (i) family history of hospital attendance due to COVID-19 and/or (ii) family history of ongoing problems due to COVID-19

The CLoCk study, described in detail elsewhere [8], is a cohort study of SARS-CoV-2 PCR-positive CYP, PCR-tested between September 2020 and March 2021 when they were aged 11 to 17 years, matched by month of test, age, sex assigned at birth, and geographical area to SARS-CoV-2 test-negative CYP using the SARS-CoV-2 testing dataset held by the United Kingdom Health Security Agency (UKHSA). After obtaining written informed consent, CYP completed an online questionnaire about their health at the time of their SARS-CoV-2 PCR test (“baseline”; retrospectively reported) and at approximately 3, 6, 12 and 24 months after their index-PCR test (with different numbers of respondents at each time point depending on the time of recruitment into the study relative to their test date). Ethical approval was provided by the Health Research Authority Yorkshire and the Humber – South Yorkshire Research Ethics Committee (REC reference: 21/YH/0060; IRAS project ID: 293,495) and the study is registered with the ISRCTN registry (ISRCTN 34804192). Here, in our main analysis, we examine the sample of original test-positive CYP who responded at 3, 6, 12 and 24 months post-infection (*N* = 943) and, in a supplementary analysis, those from the original test-positive sample who responded at 6, 12 and 24 months post-infection (*N* = 2373); see Fig. 1 for details.

### Measures

The CLoCk questionnaire included demographics, elements of the International Severe Acute Respiratory and emerging Infection Consortium (ISARIC) Paediatric COVID-19 questionnaire [13], the Mental Health of Children and Young People in England surveys [14] and, originally, 21 symptoms (mostly assessed as present/absent). Validated health scales including the EQ-5D-Y [15] (as a measure of quality of life and function) were also included. The questionnaire was largely unchanged between study enrolment and subsequent follow-ups: redundant questions (e.g. demographics and symptoms at time of testing) were removed at follow-ups, and questions on additional symptoms (e.g. sleeping difficulties) were added.

### Outcome

The Delphi research definition of PCC in CYP [9] was operationalised at the time of questionnaire completion (i.e. at all timepoints from 3 to 24 months post-infection) as experiencing ≥ 1 symptom AND problems with mobility, self-care, doing usual activities or having pain/discomfort or feeling very worried/sad, based on the EQ-5D-Y scale. CYP meeting this operationalised research definition were classified as having PCC at the time of questionnaire completion. To align our main analysis with the WHO clinical case definition of PCC in CYP [16], our main outcome of interest was defined as persistent PCC from 3 to 24 months. This meant meeting the PCC definition at 3, 6, 12 and 24 months. Our supplementary analysis examined meeting the PCC definition at 6, 12 and 24 months in a larger sample of CYP; see Fig. 1.

### Potential predictors

Pre-specified potential predictors were chosen based on their distribution in the dataset and their known association with PCC (see Table 1 for details). They included: sex (assigned at birth); age at infection (i.e. age at PCR-testing); ethnicity; region of residence; deprivation (proxied by the Index of Multiple Deprivation (IMD)); history of health conditions (specifically asthma and allergies); learning difficulties at school (pre-pandemic); having an education, health and care plan (EHCP) in place pre-pandemic, family history of hospital visits due to COVID-19 and family history of ongoing problems due to COVID-19. Sex, age, region of residence and IMD were obtained from UKHSA databases. At study enrolment, the following were self-reported: ethnicity, asthma, allergies, learning difficulties and having an EHCP. Family history of hospital visits and ongoing COVID-19 problems were self-reported for up to 24 months.

**Table 1:** Odds ratios (95% CIs) of associations between participant characteristics and persistent PCC from (a) 3 to 24 months and (b) 6 to 24 months post-infection

**Potential predictor**	**Odds ratio (95% CI) of association between potential predictor and persistent PCC**	
	**(a) Persistent PCC 3 to 24 months post-infection** (*n*= 943)	**(b) Persistent PCC 6 to 24 months post-infection **(*n* = 2373)
**Sex** **assigned at birth**		
Male	Reference	Reference
Female	1.89 (1.69, 2.12)	2.56 (1.86, 3.54)
**Age at infection (years)**		
11–14	Reference	Reference
15–17	1.58 (1.41, 1.76)	1.58 (1.21, 2.07)
**Ethnicity**		
White	Reference	Reference
Asian/Asian British	0.62 (0.53, 0.72)	1.02 (0.70, 1.47)
Black/African/Caribbean	0.49 (0.35, 0.69)	1.20 (0.56, 2.57)
Mixed	1.33 (1.09, 1.63)	1.15 (0.63, 2.10)
Other	0.79 (0.54, 1.16)	1.23 (0.42, 3.55)
Prefer not to say	-	0.66 (0.09, 5.13)
**Region of residence**		
London	Reference	Reference
East Midlands	0.49 (0.36, 0.65)	0.67 (0.38, 1.16)
East of England	1.01 (0.84, 1.22)	0.79 (0.47, 1.35)
North East	1.24 (0.92, 1.67)	0.73 (0.38, 1.41)
North West	1.18 (0.99, 1.42)	0.72 (0.44, 1.18)
South East	1.03 (0.86, 1.24)	0.83 (0.51, 1.34)
South West	2.13 (1.73, 2.62)	1.10 (0.65, 1.87)
West Midlands	1.49 (1.24, 1.78)	0.61 (0.36, 1.04)
Yorkshire and The Humber	0.63 (0.48, 0.83)	0.97 (0.59, 1.60)
**IMD **		
5 (least deprived)	Reference	Reference
4	1.10 (0.88, 1.37)	1.31 (0.87, 1.99)
3	1.73 (1.42, 2.11)	1.96 (1.31, 2.92)
2	1.72 (1.42, 2.08)	1.65 (1.09, 2.50)
1 (most deprived)	1.46 (1.21, 1.76)	1.54 (1.02, 2.34)
**History of asthma**		
No	Reference	Reference
Yes	2.05 (1.79, 2.36)	1.68 (1.17, 2.40)
**History of allergy problems **(skin eczema, hay fever, food allergies)		
No	Reference	Reference
Yes	1.92 (1.72, 2.13)	1.61 (1.24, 2.09)
**Learning difficulties at school** (pre-pandemic)		
No	Reference	Reference
Yes	6.06 (5.24, 7.01)	2.45 (1.67, 3.58)
**Education, health and care plan** (pre-pandemic)		
No	Reference	Reference
Yes	5.19 (4.43, 6.08)	2.27 (1.38, 3.75)
Family* visited hospital due to COVID-19		
No/don’t know	Reference	Reference
Yes	2.06 (1.83, 2.32)	1.74 (1.21, 2.50)
Family* has ongoing problems due to COVID-19		
No/don’t know	Reference	Reference
Yes	5.49 (4.86, 6.21)	2.91 (2.25, 3.77)

*Family defined as “family in your house”

### Sample size and missing data

We assessed whether our analytical samples were sufficiently powered to estimate the overall observed outcome probability, and how many predictor parameters could be considered before overfitting/precision became a concern [17]. We’ve used the *pmsampsize* STATA package and considered (i) small overfitting (i.e. a shrinkage factor of predictor effects ≤ 10%), (ii) small absolute difference of 0.05 in the model’s apparent and adjusted Nagelkerke’s R-squared value and (iii) precise estimation within 0.05 of the average outcome risk in the population. For the 3- to 24-month main analysis sample (*n* = 943), we assumed an outcome prevalence of 7.21% and a C-statistic of 0.75. For the 6- to 24-month supplementary analysis sample (*n* = 2373), we assumed an outcome prevalence of 11.3% and a C-statistic of 0.75. For the 3- to 24-month sample, the maximum number of parameters that could be estimated during model development was 6 with 11.33 events per candidate predictor parameter. For the 6- to 24-month sample, the maximum number of parameters that could be considered was 23; the event per candidate predictor parameter value was 11.65.

There was no missing data in our main analysis. In our supplementary analysis, 2 CYP had missing data on family history of hospital attendance and/or family history of ongoing problems due to COVID-19; they were dropped from the model-building process (Fig. 1).

### Statistical analysis

#### Characterising CYP infected with COVID-19 who persistently meet the PCC definition over a 24-month period

The prevalence of PCC at 3 months that continued at all follow-up timepoints to 24 months and at 6 months that continued to 24 months was calculated and depicted in bar charts. We also, describe the sociodemographic characteristics of the samples stratified by PCC persistence.

#### Developing and validating prediction models for persistent PCC up to 24 months post-infection

The following prediction modelling development and validation process for persistent PCC was carried out in both our main and supplementary analytical samples. Univariable associations between each potential predictor and persistent PCC were examined. Next, a multivariable logistic regression model was built using the least absolute shrinkage and selection operator (LASSO) technique to identify all potential predictors [18]. After this step, the model was further refined to ensure it had the required number of parameters (as described in the sample size calculation above). Model calibration, the agreement between observed and predicted probabilities of being classified as having persistent PCC, was assessed using calibration plots, calibration- in-the-large and calibration slope statistics [19]. Model discrimination, the ability of the model to differentiate between CYP who were classified as having persistent PCC and those who did not, was quantified using the C-statistic [19]. Internal validity of the final model was assessed using 100 bootstrap samples (drawn with replacement) [19, 20]. Confidence intervals (CIs) for the performance measures, including calibration-in-the-large, were estimated using bootstrapping. Specifically, 100 bootstrap samples were drawn with replacements from the original dataset. For each bootstrap sample, the performance measures were calculated, and the distribution of these measures across the 100 bootstrap samples was used to derive the 95% CIs. Model overfitting (optimism) was also estimated using the bootstrap samples. We calculated two shrinkage factors to adjust for overfitting: (i) a uniform shrinkage factor (i.e. the optimism-adjusted calibration slope derived using bootstrap samples) and (ii) the Heuristic shrinkage factor [21]. For use in the next step, we selected the shrinkage factor requiring the least adjustment (i.e. closest to one). The original *β* coefficients were multiplied by this shrinkage factor to obtain the optimism-adjusted coefficients; the model intercept was re-estimated based on these shrunken model coefficients to generate the final model [20, 22]. Data management and analysis were performed using STATA18. We followed guidelines by the Prognosis Research Strategy (PROGRESS) Group [23–25] and model development and validation phases followed the suggested methods. [20, 23, 26–28] The study is reported according to the Transparent Reporting of a multivariable prediction model for Individual Prognosis or Diagnosis (TRIPOD) statement [27] (see Additional File 1: Table S1).

#### Sensitivity analyses: model performance in key subgroups

We assessed, in our main analytical sample, whether the prediction was consistent across key subgroups, by determining model performance metrics (calibration slope, calibration-in-the-large and C-statistic) in subgroups stratified by age at infection (11–14, 15–17 years), sex at birth (male, female) and IMD quintiles (most-to-least deprived).

#### Role of the funding source

The funders had no role in the study design, collection, analysis, and interpretation of data, nor in the writing of the report and in the decision to submit for publication.

## Results

Nine hundred forty-three of 23,048 CYP were included in our 3-to-24-month analytical sample, while 2373 of 55,447 CYP were included in our 6-to-24-month analytical sample (Fig. 1). In general, our analytical samples contained more females and least deprived CYP compared to those invited and envisioned to be in our population (Additional File 1: Table S2).

### Characteristics of CYP meeting the PCC research definition up to 24 months post-infection

**Fig. 2:**
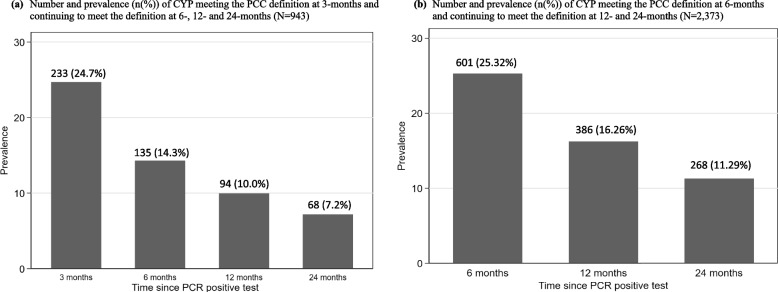
Number and prevalence (*n* (%)) of CYP continuing to meet the PCC definition over time. NB: the prevalence of persistent PCC up to 24 months (shown as the bar at 24 months since a PCR positive test) is the outcome that is predicted in the subsequent models

While 25% (233/943) of CYP met the research definition of PCC at 3 months post-infection, only 7% (68/943) continued to meet the PCC definition persistently to 24 months (Fig. 2, Additional File 1: Table S3). Those who met the PCC definition persistently from 3 to 24 months were more likely to be female (vs. male), older at infection (vs. younger), live in more (vs. least) deprived areas, have a history of asthma, allergy problems, learning difficulties at school, an EHCP, a family history of hospital visits or ongoing problems due to COVID-19 (Table 1, Additional File 1: Table S3). For example, the odds ratio (OR) of meeting the PCC definition at 3, 6, 12 and 24 months was 1.58 (95% CI: 1.41, 1.76) comparing CYP who were 15 to 17 years old at infection to those who were 11 to 14 years old. There were also differences with respect to ethnicity and region of residence. In the supplemental analysis, while 25% (601/2373) met the PCC definition at 6 months post-infection, 11% (268/2373) of CYP continued to meet the PCC definition at all subsequent timepoints, i.e. at 12 and 24 months (Fig. 2, Additional File 1: Table S3). Those who met the PCC definition at all these timepoints had a broadly similar profile to the sub-sample meeting the PCC definition at 3, 6, 12 and 24 months. For example, the OR of meeting the PCC definition at 6, 12 and 24 months was 1.58 (95% CI: 1.21, 2.07) comparing CYP who were 15 to 17 years old to CYP who were 11 to 14 years old at infection.

### Model development

The final model, predicting persistent PCC from 3 to 24 months included sex, history of asthma, allergy, learning difficulties at school and family history of ongoing problems due to COVID-19 (Additional File 1: Table S4). For the final model predicting persistent PCC from 6 to 24 months, additional predictors were age, region of residence, IMD, an EHCP and family history of hospital visits due to COVID-19 (Additional File 1: Table S4).

### Model validation

**Fig. 3:**
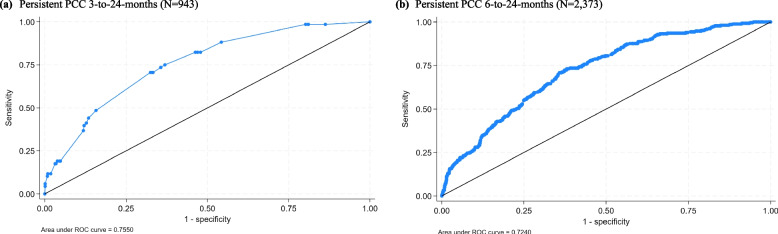
Area under the curve for the final shrunken models

The original predictive model was well calibrated in the model development data to predict persistent PCC from 3 to 24 months, with an apparent calibration slope of 1.000 (95% CI: 0.999, 1.000) and an apparent calibration-in-the-large of 0.000 (95% CI: − 0.000, 0.000; Additional File 1: Table S5). The discrimination was also good, with a C-statistic of 0.757 (95% CI: 0.699, 0.814). The bootstrapping approach provided a shrinkage factor of 0.9398; the heuristic shrinkage factor was 0.9139. We used the bootstrapping shrinkage factor as it required a smaller adjustment and applied it to the original β coefficients to obtain the optimism-adjusted coefficients before re-estimating the intercept for the final model given in Additional File 1: Table S6. The final shrunken predictive model showed good overall model calibration: confirmed by the calibration-in-the-large (0.000), calibration slope (1.064) and a calibration plot showing narrow confidence intervals and closely aligned predicted and observed probabilities for 10 equally sized risk groups (Additional File 1: Fig. S1). It also showed moderate-to-strong discrimination with a C-statistic of 0.755 (Table 2, Fig. 3a).

**Table 2:** Model performance statistics of the original/final shrunken models

Measure	Original model	Shrunken model
**Predicting persistent PCC 3 to 24 months post-infection**		
Calibration slope^a^	1.000 (0.730, 1.269)	1.064 (0.777,1.350)
Calibration in the large^a^	0.000 (− 0.257, 0.257)	0.000 (− 0.258,0.258)
C Statistic^c^	0.756 (0.699, 0.810)	0 .755 (0.698,0.811)
**Predicting persistent PCC 6 to 24 months post-infection**		
Calibration slope^a^	1.000 (0.834, 1.165)	1.142 (0.953, 1.331)
Calibration in the large^b^	0.006 (− 0.126, 0.138)	0.005 (− 0.125, 0.136)
C Statistic^c^	0.726 (0.694, 0.755)	0.724 (0.693, 0.756)

^a^A measure of calibration; values closer to one indicate better calibration

^b^A measure of calibration; values closer to zero indicate better calibration

^c^A measure of discrimination; values closer to one indicate stronger discrimination

In the model development data predicting persistent PCC from 6 to 24 months, the original model was well calibrated (calibration slope = 1.000 (95% CI: 0.999, 1.000); calibration-in-the-large = 0.000 (95% CI: − 0.000, 0.000; Additional File 1: Table S5) and showed good discrimination (C-statistic = 0.737 (95% CI: 0.698, 0.766). The bootstrapping approach provided a shrinkage factor of 0.8755; the heuristic shrinkage factor was 0.8725. Using the bootstrapping shrinkage factor we obtained the optimism-adjusted coefficients given in Additional File 1: Table S6. The final shrunken predictive model showed strong discrimination (C-statistic = 0.724; Table 2, Fig. 3b); and, the final shrunken model also showed good overall calibration (calibration slope = 1.142; calibration-in-the-large = 0.005; Table 2).

### Sensitivity analyses

Model performance statistics in age, sex at birth and IMD subgroups demonstrated broadly good calibration and discrimination in all subgroups, with the exception of the least deprived IMD group (Additional File 1: Table S7). For example, the C-statistic for age at infection, sex at birth and IMD quintiles 1 to 4 subgroups, ranged from 0.705 to 0.847. In contrast, IMD quintile 5 (i.e. least deprived) had a C-statistic of 0.629.

### Worked examples

We demonstrate with hypothetical examples the predicted risk of persistent PCC in Table 3. As an example, the predicted risk of persistent PCC from 3 to 24 months post-infection for a hypothetical male, with a history of asthma and a family history of ongoing problems due to COVID-19 was 8.1% (worked example 3). If a similar boy also had learning difficulties at school pre-pandemic, it would be 22.8% (worked example 4).

**Table 3:**
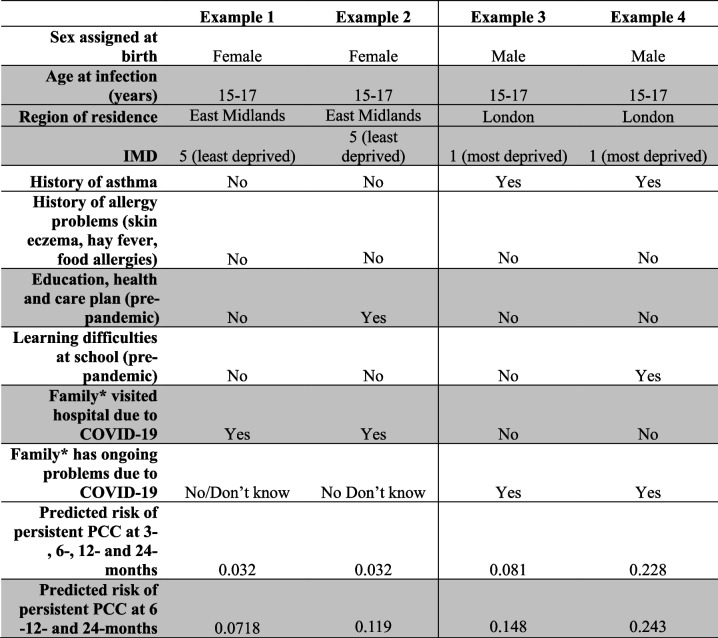
Hypothetical examples of predicted risk of persistent PCC, using our prediction models

^*^Family defined as “family in your house”

Variables highlighted in grey are only used as predictors in the persistent PCC 6 to 24 months post-infection model. Hence examples 1 and 2 have the same predicted risk of persistent PCC 3 to 24 months (i.e. the only characteristic that differs between examples 1 and 2 [having an education, health and care plan pre-pandemic] is not used to predict risk of persistent PCC 3 to 24 months). In contrast, predict risk of persistent PCC 6 to 24 months and 3 to 24 months differs for examples 3 and 4 because the characteristic that differs between the examples (learning difficulties at school pre-pandemic) is used in both prediction models

## Discussion

This study shows two key findings. Firstly, the data show that many CYP who initially meet the research definition of PCC get better over time. For example, while 25% of CYP met the PCC definition 3 months post-infection, only 7% of CYP continued to meet this definition at 6, 12 and 24 months post-infection. While acknowledging that SARS-CoV-2 infection can have a long-lasting impact on some CYP [4], our finding is in line with previous reports from the CLoCk study [10], and elsewhere [29] and demonstrates that post-infection symptoms in CYP generally improve over time. Second, in our final models, we found that female sex, history of asthma, allergy problems, learning difficulties at school and family history of ongoing COVID-19 problems all predicted persistent PCC from 3 to 24 months and from 6 to 24 months. In addition, older age at the time of infection, living in London or the South West, living in more deprived areas at the time of infection, being allocated an EHCP pre-pandemic and having a family history of hospital visits due to COVID-19 also predicted persistent PCC from 6 to 24 months. To our knowledge, these are the only prediction models estimating the risk of CYP persistently meeting the PCC definition up to 24 months post-infection. The models could be used to triage CYP early after infection to identify those who may benefit from earlier targeted support.

We acknowledge the study limitations. The focus of our study was on model development and internal validation, and therefore we did not conduct external validation in this study. However, external validation is recommended prior to clinical application of these models. In particular, it is important to externally validate these models in different settings and populations [30]. For example, these models can be validated in cohorts of CYP after infection by other variants (for example, the Omicron variant [31]). In addition to the above, selection bias may exist in our study. Approximately 4% of the target population of invited test positives were part of the examined analytical samples, and these CYP may not be representative of the broader population of CYP who PCR-tested positive for COVID-19 in England, between September 2020 and March 2021. Moreover, our models have been developed based on infection in CYP by wild-type or the Alpha (B.1.1.7) variant and may not be transferable to other more recent variants. However, previous studies suggest that post-infection symptom profiles are similar for different variants [11]. Nevertheless, as variant, background infection and vaccination rates differ, so too might predictors of persistent PCC. Therefore, as part of updating and externally validating the models developed here, it is important to apply them in cohorts of CYP that have been infected during subsequent COVID-19 infection waves and have been followed up for (at least) 24 months, when such data become available. Importantly, due to changes in COVID-19 testing policies in England over time, we were unable to account sufficiently for subsequent reinfections in our analytical samples. Therefore, while we are certain of the CYP’s PCR-positive status at baseline, we acknowledge we cannot accurately distinguish whether subsequent symptoms were due to acute reinfection or persistent symptoms from the original (or subsequent) infection. We were also restricted in terms of the potential predictors examined for two reasons. First, because CYP enrolled into our study 3 months post-index-infection, we did not use information that could be subject to recall bias, for example, self-reported physical/mental health at the time of infection. Second, we were limited by questions asked in the CLoCk questionnaire (for example, we did not have information on the severity of index infection), and this may further compromise our prediction model. However, we did use available questionnaire information on variables deemed less likely to be subject to recall bias, for example, having an EHCP in place pre-pandemic. We also used variables collected post-baseline that were likely to be relevant (but were perhaps less prevalent at the time of study enrolment) for example, family history of ongoing problems due to COVID-19. PCC prevalence estimates in CYP vary greatly, with systematic reviews reporting prevalences ranging from 3.67% to 66.49% [32, 33]. Thus, there was no external, gold-standard prevalence to use in our sample size calculations and we used outcome prevalences based on our analytical samples. In addition, our sample size for predicting persistent PCC from 3 to 24 months was relatively small (*n* = 943) and this had implications in terms of the number of predictors we could include in the model and, therefore, on model performance. Nonetheless, both our final models had good predictive ability, calibration and discrimination. While we are able to characterise and predict PCC up to 24 months post-infection, extending our previous work [12], we are unable to say with confidence whether PCC was present (or not) between data collection sweeps. It may be that our operationalisation of the PCC definition has been too inclusive. However, in the absence of an objective biomarker of PCC, we have had to rely on the consensus Delphi-research definition of PCC [9], which broadly aligns with the WHO definition [16] with the important exception that the latter requires symptoms to have arisen within 3 months from infection. It is for this reason that we developed, and placed most emphasis on, a model predicting persistent PCC from 3 to 24 months, despite data only being available for a small sub-sample at all these timepoints. Finally, as with any prediction model, caution is required for predictions based on data extrapolation/situations where there are only a very small number of observations for different predictor combinations.

## Conclusions

Understanding which CYP are at risk of experiencing persistent PCC for months/years after infection is important for decision-making and risk management by the individual, their families and care providers. Using data from a large national cohort study of CYP, we update and extend our previously developed prediction model for experiencing persistent PCC for up to 24 months post-infection [12]. Further studies to determine the clinical and pathophysiological phenotype of PCC are warranted. In the interim, while our models need external validation (in different datasets, countries, etc.), we hope that they will eventually serve as a useful tool for the early identification and management of CYP at risk of persistent PCC.

## Supplementary Information

Additional File 1: Table S1: TRIPOD checklist for prognostic model development and validation studies. Table S2: Participant characteristics of (a) all invited to enrol into the study at 3-months post-positive PCR-test, (b) those included in the 3-to-24-month analytical sample, (c) all envisioned to take part in the study at 6-months post-positive PCR-test and (d) those included in the 6-to-24-month analytical sample. Table S3: Study participant characteristics, stratified by persistent PCC 3-to-24-months and 6-to-24-months. Table S4: Final multivariable analysis developed models (original coefficients). Table S5: Model performance statistics during internal validation (using 100 bootstrap samples). Table S6: Final model coefficients after adjusting for overfitting. Table S7: Model performance statistics of the final shrunken models in key subgroups (3-to-24-month sample). Figure S1: Calibration plots.

## Data Availability

Data is not publicly available. All requests for data will be reviewed by the Children & young people with Long COVID (CLoCk) study team, to verify whether the request is subject to any intellectual property or confidentiality obligations. Requests for access to the participant-level data from this study can be submitted via email to ich.clock@ucl.ac.uk with detailed proposals for approval. A signed data access agreement with the CLoCk team is required before accessing shared data. Code is not made available as we have not used custom code or algorithms central to our conclusions.

## Abbreviations

CYP: Children and young people
CIs: Confidence intervals
EHCP: Education, health and care plan
IMD: Index of Multiple Deprivation
ISARIC: International Severe Acute Respiratory and emerging Infection Consortium
LASSO: Least absolute shrinkage and selection operator
PCC: Post-COVID-19 condition
PROGRESS: Prognosis Research Strategy
TRIPOD: Transparent Reporting of a multivariable prediction model for Individual Prognosis or Diagnosis
UKHSA: United Kingdom Health Security Agency
