# Supplementary material for: Predicting post-COVID-19 condition in children and young people up to 24 months after a positive SARS-CoV-2 PCR-test: the CLoCk study
Source: BMC Med. 2024 Nov 7;22:520. doi: 10.1186/s12916-024-03708-1 (PMC11545583; doi:10.1186/s12916-024-03708-1)
Supplement: Supplementary file 1 — Additional File 1: Table S1: TRIPOD checklist for prognostic model development and validation studies. Table S2: Participant characteristics of (a) all invited to enrol into the study at 3-months post-positive PCR-test, (b) those included in the 3-to-24-month analytical sample, (c) all envisioned to take part in the study at 6-months post-positive PCR-test and (d) those included in the 6-to-24-month analytical sample. Table S3: Study participant characteristics, stratified by persistent PCC 3-to-24-months and 6-to-24-months. Table S4: Final multivariable analysis developed models (original coefficients). Table S5: Model performance statistics during internal validation (using 100 bootstrap samples). Table S6: Final model coefficients after adjusting for overfitting. Table S7: Model performance statistics of the final shrunken models in key subgroups (3-to-24-month sample). Figure S1: Calibration plots. [file 12916_2024_3708_MOESM1_ESM.docx]

**Additional File 1: Tables and Figures**

**Additional File 1: Table S1: TRIPOD checklist for prognostic model development and validation studies**

| **Section/Topic** | **Item** |  | **Checklist Item** | **Page** |
| --- | --- | --- | --- | --- |
| **Title and abstract** | | | | |
| Title | 1 | D;V | Identify the study as developing and/or validating a multivariable prediction model, the target population, and the outcome to be predicted. | 1 |
| Abstract | 2 | D;V | Provide a summary of objectives, study design, setting, participants, sample size, predictors, outcome, statistical analysis, results, and conclusions. | 2 |
| **Introduction** | | | | |
| Background and objectives | 3a | D;V | Explain the medical context (including whether diagnostic or prognostic) and rationale for developing or validating the multivariable prediction model, including references to existing models. | 3-4 |
|  | 3b | D;V | Specify the objectives, including whether the study describes the development or validation of the model or both. | 4 |
| **Methods** | | | | |
| Source of data | 4a | D;V | Describe the study design or source of data (e.g., randomized trial, cohort, or registry data), separately for the development and validation data sets, if applicable. | 4 |
|  | 4b | D;V | Specify the key study dates, including start of accrual; end of accrual; and, if applicable, end of follow-up. | 4 |
| Participants | 5a | D;V | Specify key elements of the study setting (e.g., primary care, secondary care, general population) including number and location of centres. | 4-5 |
|  | 5b | D;V | Describe eligibility criteria for participants. | 4-5 |
|  | 5c | D;V | Give details of treatments received, if relevant. | N/A |
| Outcome | 6a | D;V | Clearly define the outcome that is predicted by the prediction model, including how and when assessed. | 5 |
|  | 6b | D;V | Report any actions to blind assessment of the outcome to be predicted. | N/A |
| Predictors | 7a | D;V | Clearly define all predictors used in developing or validating the multivariable prediction model, including how and when they were measured. | 5 |
|  | 7b | D;V | Report any actions to blind assessment of predictors for the outcome and other predictors. | N/A |
| Sample size | 8 | D;V | Explain how the study size was arrived at. | 5-6 |
| Missing data | 9 | D;V | Describe how missing data were handled (e.g., complete-case analysis, single imputation, multiple imputation) with details of any imputation method. | 5-6 |
| Statistical analysis methods | 10a | D | Describe how predictors were handled in the analyses. | 5-6 |
|  | 10b | D | Specify type of model, all model-building procedures (including any predictor selection), and method for internal validation. | 6-7 |
|  | 10c | V | For validation, describe how the predictions were calculated. | 6-7 |
|  | 10d | D;V | Specify all measures used to assess model performance and, if relevant, to compare multiple models. | 6-7 |
|  | 10e | V | Describe any model updating (e.g., recalibration) arising from the validation, if done. | N/A |
| Risk groups | 11 | D;V | Provide details on how risk groups were created, if done. | N/A |
| Development vs. validation | 12 | V | For validation, identify any differences from the development data in setting, eligibility criteria, outcome, and predictors. | 6-7 |
| **Results** | | | | |
| Participants | 13a | D;V | Describe the flow of participants through the study, including the number of participants with and without the outcome and, if applicable, a summary of the follow-up time. A diagram may be helpful. | Figure 1 |
|  | 13b | D;V | Describe the characteristics of the participants (basic demographics, clinical features, available predictors), including the number of participants with missing data for predictors and outcome. | 7-8 |
|  | 13c | V | For validation, show a comparison with the development data of the distribution of important variables (demographics, predictors and outcome). | N/A |
| Model development | 14a | D | Specify the number of participants and outcome events in each analysis. | 7-8 |
|  | 14b | D | If done, report the unadjusted association between each candidate predictor and outcome. | Table 1/ Supplement Table 3 |
| Model specification | 15a | D | Present the full prediction model to allow predictions for individuals (i.e., all regression coefficients, and model intercept or baseline survival at a given time point). | Supplement Table S6 |
|  | 15b | D | Explain how to the use the prediction model. | 9 |
| Model performance | 16 | D;V | Report performance measures (with CIs) for the prediction model. | 8-9 |
| Model-updating | 17 | V | If done, report the results from any model updating (i.e., model specification, model performance). | N/A |
| **Discussion** | | | | |
| Limitations | 18 | D;V | Discuss any limitations of the study (such as nonrepresentative sample, few events per predictor, missing data). | 10-11 |
| Interpretation | 19a | V | For validation, discuss the results with reference to performance in the development data, and any other validation data. | N/A |
|  | 19b | D;V | Give an overall interpretation of the results, considering objectives, limitations, results from similar studies, and other relevant evidence. | 11 |
| Implications | 20 | D;V | Discuss the potential clinical use of the model and implications for future research. | 11 |
| **Other information** | | | | |
| Supplementary information | 21 | D;V | Provide information about the availability of supplementary resources, such as study protocol, Web calculator, and data sets. | Supplement Files |
| Funding | 22 | D;V | Give the source of funding and the role of the funders for the present study. | 13 |

*Items relevant only to the development of a prediction model are denoted by D, items relating solely to a validation of a prediction model are denoted by V, and items relating to both are denoted D;V.

**Additional File 1: Table S2: Participant characteristics of (a) all invited to enrol into the study at 3-months post-positive PCR-test, (b) those included in the 3-to-24-month analytical sample, (c) all envisioned to take part in the study at 6-months post-positive PCR-test and (d) those included in the 6-to-24-month analytical sample**

|  | 1. Invited to enrol at 3-months post-positive PCR-test (N=23,048) | 1. Included in the 3-to-24-month analytical sample (N=943) |
| --- | --- | --- |
| % included in analytical sample |  | 4.09% |
| **Sex assigned at birth**  Male  Female | 10,636 (46.15)  12,412 (53.85) | 298 (31.60)  645 (68.40) |
| **Age at infection (years)**  11-14  15-17 | 10,651 (46.21)  12,397 (53.79) | 401 (42.52)  542 (57.48) |
| **Region of residence**  London  East Midlands  East of England  North East  North West  South East  South West  West Midlands  Yorkshire and The Humber | 4,412 (19.14)  1,815 (7.87)  3,392 (14.72)  819 (3.55)  3,235 (14.04)  3,496 (15.17)  1,238 (5.37)  2,854 (12.38)  1,787 (7.75) | 137 (14.53)  92 (9.76)  139 (14.74)  31 (3.29)  111 (11.77)  169 (17.92)  88 (9.33)  118 (12.51)  58 (6.15) |
| **IMD**  5 (least deprived)  4  3  2  1 (most deprived) | 3,280 (14.23)  3,679 (15.96)  4,159 (18.04)  5,198 (22.55)  6,732 (29.21) | 224 (23.75)  188 (19.94)  177 (18.77)  187 (19.83)  167 (17.71) |
|  | (c) Envisioned to take part at 6-months post-positive PCR-test (N=55,447) | (d) Included in the 6-to-24-month analytical sample (N=2,373) |
| % included in analytical sample |  | 4.28% |
| **Sex assigned at birth**  Male  Female | 26,004 (46.90)  29,443 (53.10) | 816 (34.39)  1,557 (65.61) |
| **Age at infection (years)**  11-14  15-17 | 26,757 (48.26)  28,690 (51.74) | 1,035 (43.62)  1,338 (56.38) |
| **Region of residence**  London  East Midlands  East of England  North East  North West  South East  South West  West Midlands  Yorkshire and The Humber | 7,950 (14.34)  4,771 (8.60)  5,546 (10.00)  3,079 (5.55)  10,363 (18.69)  6,816 (12.29)  2,934 (5.29)  7,386 (13.32)  6,602 (11.91) | 258 (10.87)  243 (10.24)  253 (10.66)  136 (5.73)  356 (15.00)  348 (14.66)  197 (8.30)  309 (13.02)  273 (11.50) |
| **IMD**  5 (least deprived)  4  3  2  1 (most deprived) | 8,720 (15.73)  9,112 (16.43)  9,589 (17.29)  11,528 (20.79)  16,498 (29.75) | 581 (24.48)  502 (21.15)  429 (18.08)  418 (17.61)  443 (18.67) |

**Additional File 1: Table S3: Study participant characteristics, stratified by persistent PCC 3-to-24-months and 6-to-24-months**

| Potential Predictor | Persistent PCC | | | |
| --- | --- | --- | --- | --- |
|  | 3-to-24-months  (n= 943) | | 6-to-24-months  (n= 2,373) | |
|  | No | Yes | No | Yes |
| Prevalence | 875 (92.79) | 68 (7.21) | 2,105 (88.71) | 268 (11.29) |
| **Sex assigned at birth**  Male  Female | 286 (95.97)  589 (91.32) | 12 (4.03)  56 (8.68) | 767 (94.00)  1,338 (85.93) | 49 (6.00)  219 (14.07) |
| **Age at infection (years)**  11-14  15-17 | 382 (95.26)  493 (90.96) | 19 (4.74)  49 (9.04) | 944 (91.21)  1,161 (86.77) | 91 (8.79)  177 (13.23) |
| **Ethnicity**  White  Asian/Asian British  Black/African/Caribbean  Mixed  Other  Prefer not to say | 643 (92.12)  141 (95.27)  33 (97.06)  38 (90.48)  15 (93.75)  5 (100.00) | 55 (7.88)  7 (4.73)  1 (2.94)  4 (9.52)  1 (6.25)  0 (0.00) | 1,626 (88.85)  298 (88.69)  53 (86.89)  90 (87.38)  26 (86.67)  12 (92.31) | 204 (11.15)  38 (11.31)  8 (13.11)  13 (12.62)  4 (13.33)  1 (7.69) |
| **Region of residence**  London  East Midlands  East of England  North East  North West  South East  South West  West Midlands  Yorkshire and The Humber | 125 (91.24)  88 (95.65)  129 (92.81)  30 (96.77)  105 (94.59)  156 (92.31)  76 (86.36)  110 (93.22)  56 (96.55) | 12 (8.76)  4 (4.35)  10 (7.19)  1 (3.23)  6 (5.41)  13 (7.69)  12 (13.64)  8 (6.78)  2 (3.45) | 223 (86.43)  220 (90.53)  225 (88.93)  122 (89.71)  320 (89.89)  308 (88.51)  168 (85.28)  282 (91.26)  237 (86.81) | 35 (13.57)  23 (9.47)  28 (11.07)  14 (10.29)  36 (10.11)  40 (11.49)  29 (14.72)  27 (8.74)  36 (13.19) |
| **IMD**  5 (least deprived)  4  3  2  1 (most deprived) | 212 (94.64)  176 (93.62)  159 (89.83)  171 (91.44)  157 (94.01) | 12 (5.36)  12 (6.38)  18 (10.17)  16 (8.56)  10 (5.99) | 534 (91.91)  450 (89.64)  366 (85.31)  365 (87.32)  390 (88.04) | 47 (8.09)  52 (10.36)  63 (14.69)  53 (12.68)  53 (11.96) |
| **History of asthma**  No  Yes | 796 (93.65)  79 (84.95) | 54 (6.35)  14 (15.05) | 1,895 (89.34)  210 (83.33) | 226 (10.66)  42 (16.67) |
| **History of allergy problems** (skin eczema, hay fever, food allergies)  No  Yes | 602 (94.80)  273 (88.64) | 33 (5.20)  35 (11.36) | 1,457 (90.33)  648 (85.26) | 156 (9.67)  112 (14.74) |
| **Learning difficulties at school** (pre-pandemic)  No  Yes | 835 (95.43)  40 (4.57) | 55 (80.88)  13 (19.12) | 1,968 (93.49)  137 (6.51) | 229 (85.45)  39 (14.55) |
| **Education, health and care plan** (pre-pandemic)  No  Yes | 848 (93.50)  27 (75.00) | 59 (6.50)  9 (25.00) | 2,029 (89.15)  76 (78.35) | 247 (10.85)  21 (21.65) |
| **Family* visited hospital due to COVID-19**  No / Don’t know  Yes | 749 (93.28)  126 (90.00) | 54 (6.72)  14 (10.00) | 1,907 (89.36)  198 (82.85) | 227 (10.64)  41 (17.15) |
| **Family* has ongoing problems due to COVID-19**  No / Don’t know  Yes | 537 (96.58)  338 (87.34) | 19 (3.42)  49 (12.66) | 1,505 (92.39)  600 (80.65) | 124 (7.61)  144 (19.35) |

*family defined as “family in your house”

**Additional File 1: Table S4: Final multivariable analysis developed models (original coefficients)**

|  | Predicting persistent PCC 3-to-24-months | Predicting persistent PCC 6-to-24-months |
| --- | --- | --- |
| **Sex assigned at birth** |  |  |
| Male | 0 | 0 |
| Female | 0.8525512 | 0.9218136 |
| **Age at infection (years)** |  |  |
| 11-14 |  | 0 |
| 15-17 |  | 0.3048007 |
| **Region of residence** |  |  |
| London |  | 0 |
| East Midlands |  | -0.4090268 |
| East of England |  | -0.2743838 |
| North East |  | -0.3446971 |
| North West |  | -0.380946 |
| South East |  | -0.2373241 |
| South West |  | 0.1342224 |
| West Midlands |  | -0.6061774 |
| Yorkshire and The Humber |  | -0.0247977 |
| **IMD** |  |  |
| 5 (least deprived) |  | 0 |
| 4 |  | 0.1198349 |
| 3 |  | 0.6057013 |
| 2 |  | 0.4828662 |
| 1 (most deprived) |  | 0.4030898 |
| **History of asthma** |  |  |
| No | 0 | 0 |
| Yes | 0.6749611 | 0.2962728 |
| History of allergy problems  (skin eczema, hay fever, food allergies) |  |  |
| No | 0 | 0 |
| Yes | 0.5917628 | 0.3454753 |
| Education, health and care plan  (pre-pandemic) |  |  |
| No |  | 0 |
| Yes |  | 0.6347771 |
| Learning difficulties at school  (pre-pandemic) |  |  |
| No | 0 | 0 |
| Yes | 1.295874 | 0.7038527 |
| Family* visited hospital due to COVID-19 |  |  |
| No/Don’t know |  | 0 |
| Yes |  | 0.2031328 |
| Family* has ongoing problems due to COVID-19 |  |  |
| No/Don’t know | 0 | 0 |
| Yes | 1.224480 | 0.9385513 |
| **Constant term** | -4.348078 | -3.673141 |

*family defined as “family in your house”

**Additional File 1: Table S5: Model performance statistics during internal validation (using 100 bootstrap samples)**

| Measure | Apparent performance of the model  Mean (Standard Deviation) |  | Average optimism | Optimism corrected performance | |
| --- | --- | --- | --- | --- | --- |
|  | **Predicting PCC 3-to-24-months post-infection** | | | |  |
| Calibration slope* | 1.00000 (0.0000) |  | 0.06014 | 0.93986 | |
| Calibration in the large** | 0.00000 (0.0000) |  | -0.02271 | -0.02271 | |
| C Statistic*** | 0.75672 (0.02940) |  | 0.01149 | 0.74451 | |
|  | **Predicting PCC 6-to-24-months post-infection** | | | |  |
| Calibration slope* | 1.0000 (0.0000) |  | 0.12450 | 0.87550 | |
| Calibration in the large** | 0.0000 (0.0000) |  | 0.00263 | -0.00263 | |
| C Statistic*** | 0.73662 (0.0149) |  | 0.02449 | 0.69951 | |

*A measure of calibration; values closer to one indicate better calibration

**A measure of calibration; values closer to zero indicate better calibration

***A measure of discrimination; values closer one indicate stronger discrimination

**Additional File 1: Table S6: Final model coefficients after adjusting for overfitting**

|  | Predicting PCC 3-to-24-months post-infection | Predicting PCC 6-to-24-months post-infection |
| --- | --- | --- |
| **Sex assigned at birth** |  |  |
| Male | 0 | 0 |
| Female | 0.801398 | 0.807048 |
| **Age at infection (years)** |  |  |
| 11-14 |  | 0 |
| 15-17 |  | 0.266853 |
| **Region of residence** |  |  |
| London |  | 0 |
| East Midlands |  | -0.358103 |
| East of England |  | -0.240223 |
| North East |  | -0.301782 |
| North West |  | -0.333518 |
| South East |  | -0.207777 |
| South West |  | 0.117512 |
| West Midlands |  | -0.530708 |
| Yorkshire and The Humber |  | -0.021710 |
| **IMD** |  |  |
| 5 (least deprived) |  | 0 |
| 4 |  | 0.104915 |
| 3 |  | 0.530291 |
| 2 |  | 0.422749 |
| 1 (most deprived) |  | 0.352905 |
| **History of asthma** |  |  |
| No | 0 | 0 |
| Yes | 0.634463 | 0.259387 |
| History of allergy problems  (skin eczema, hay fever, food allergies) |  |  |
| No | 0 | 0 |
| Yes | 0.556257 | 0.302464 |
| Education, health and care plan  (pre-pandemic) |  |  |
| No |  | 0 |
| Yes |  | 0.555747 |
| Learning difficulties at school  (pre-pandemic) |  |  |
| No | 0 | 0 |
| Yes | 1.218122 | 0.616223 |
| Family* visited hospital due to COVID-19 |  |  |
| No/Don’t know |  | 0 |
| Yes |  | 0.177843 |
| Family* has ongoing problems due to COVID-19 |  |  |
| No/Don’t know | 0 | 0 |
| Yes | 1.151011 | 0.821702 |
| **Constant term** | -4.22079 | -3.45213 |

*family defined as “family in your house”

| **Additional File 1: Table S7: Model performance statistics of the final shrunken models in key subgroups**  **(3-to-24-month sample)** | | |
| --- | --- | --- |
|  | **Predicting PCC 3-to-24-months post-infection** | |
|  | Measure | Shrunken Model |
| **Age at infection (years)** |  |  |
| 11-14 (n=401) | Calibration slope* | 1.211 (0.666, 1.756) |
|  | Calibration in the large** | -0.363 (-0.837, 0.111) |
|  | C Statistic*** | 0.814 (0.749, 0.878) |
| 15-17 (n=542) | Calibration slope* | 0.977 (0.639, 1.314) |
|  | Calibration in the large** | 0.193 (-0.114, 0.499) |
|  | C Statistic*** | 0.726 (0.652, 0.800) |
| **Sex assigned at birth** |  |  |
| Male (n=298) | Calibration slope* | 1.312 (0.680, 1.944) |
|  | Calibration in the large** | -0.033 (-0.627, 0.559) |
|  | C Statistic*** | 0.806 (0.677, 0.936) |
| Female (n=645) | Calibration slope* | 1.000 (0.651, 1.326) |
|  | Calibration in the large** | 0.007 (-0.276, 0.291) |
|  | C Statistic*** | 0.710 (0.640, 0.779) |
| **IMD** |  |  |
| 1 [most deprived] (n=167) | Calibration slope* | 1.240 (0.398, 2.082) |
|  | Calibration in the large** | -0.145 (-0.800, 0.510) |
|  | C Statistic*** | 0.779 (0.614, 0.945) |
| 2 (n=187) | Calibration slope* | 1.515 (0.821, 2.210) |
|  | Calibration in the large** | 0.251 (-0.285, 0.787) |
|  | C Statistic*** | 0.847 (0.768, 0.926) |
| 3 (n=177) | Calibration slope* | 0.837 (0.297, 1.377) |
|  | Calibration in the large** | 0.325 (-0.187, 0.837) |
|  | C Statistic*** | 0.705 (0.586, 0.823) |
| 4 (n=188) | Calibration slope* | 1.398 (0.654, 2.142) |
|  | Calibration in the large** | -0.231 (-0.836, 0.374) |
|  | C Statistic*** | 0.823 (0.729, 0.917) |
| 5 [least deprived] (n=224) | Calibration slope* | 0.533 (-0.101, 1.166) |
|  | Calibration in the large** | -0.276 (-0.875, 0.323) |
|  | C Statistic*** | 0.629 (0.481, 0.776) |

*A measure of calibration; values closer to one indicate better calibration

**A measure of calibration; values closer to zero indicate better calibration

***A measure of discrimination; values closer to one indicate stronger discrimination

| **Additional File 1: Figure S1: Calibration plots** | |
| --- | --- |
| **(a)** Predicting PCC at 3-to-24-months post-infection | **(b)** Predicting PCC at 6-to-24-months post-infection |
| 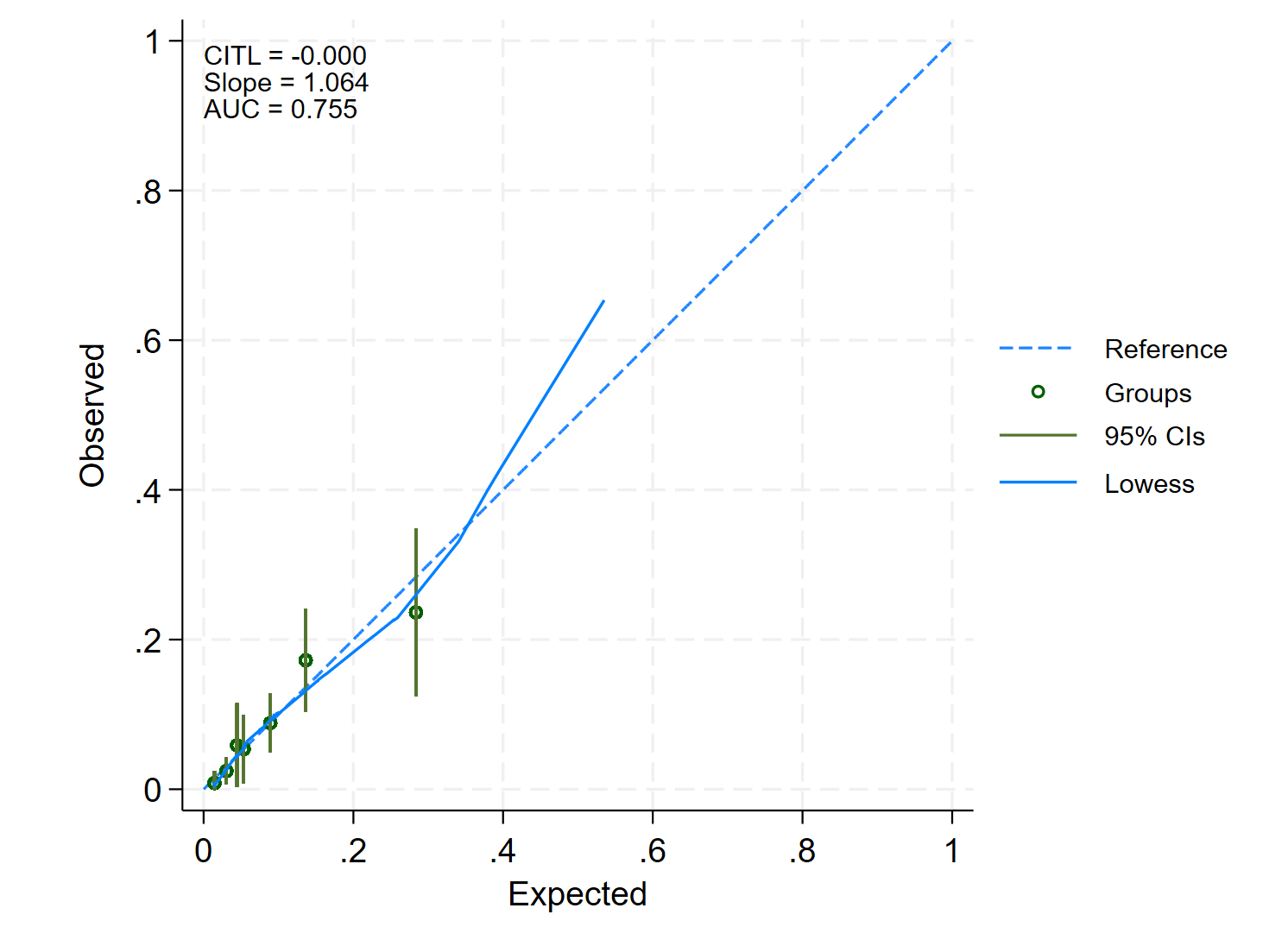 | 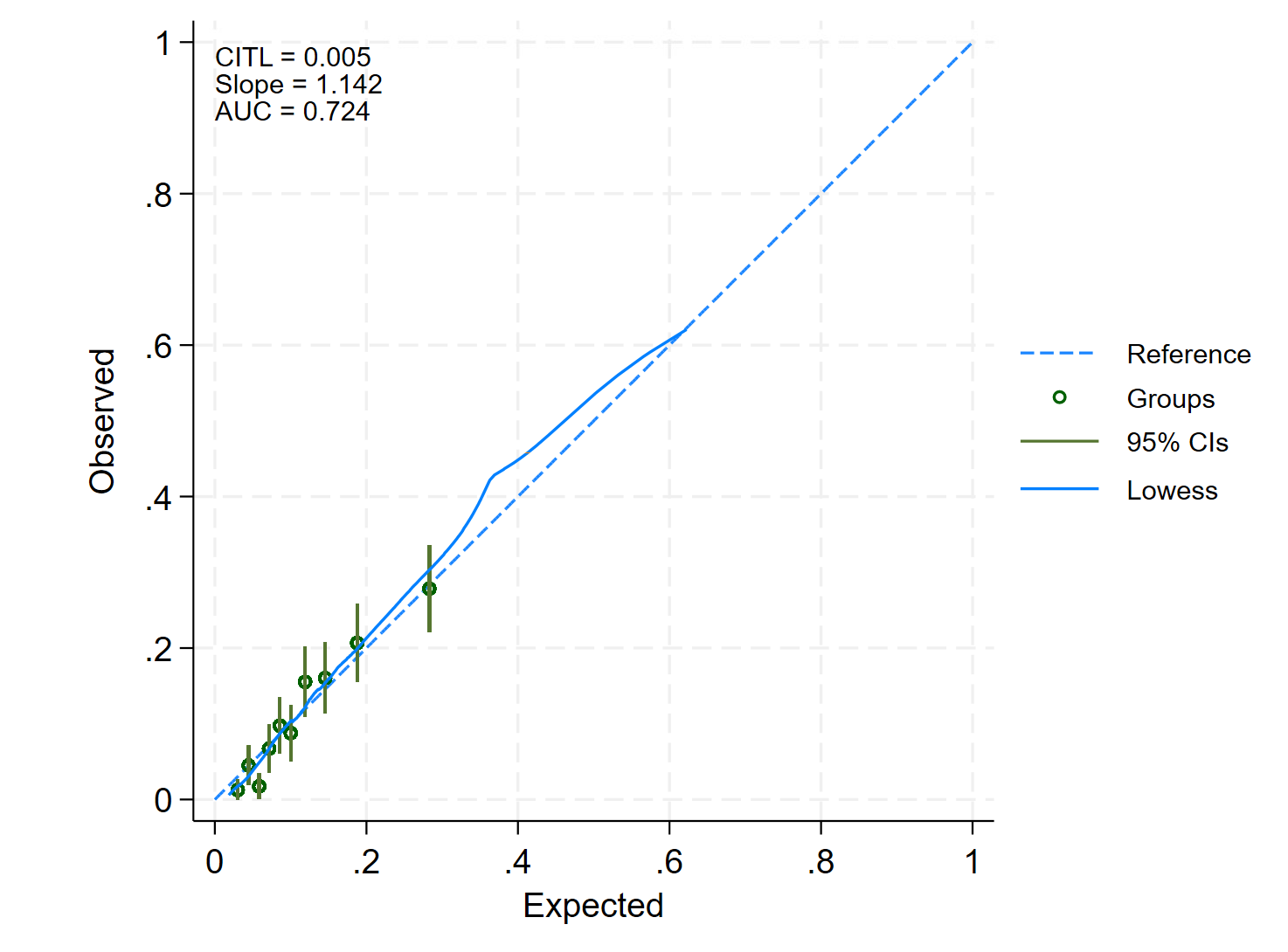 |
| Observed and predicted risk of persistent PCC after a positive PCR test. The graphs show the mean predicted probability (hollow dots) and 95% confidence intervals of presistent PCC plotted against the observed proportion with persistent PCC for 10 equally sized groups. The dashed line represents the line of equality and perfect calibration. The blue solid line is a smoothed locally weighted scatter plot smoothing (Lowess) regression line. | |
